# Dynamic Gene Network Analysis of Caco-2 Cell Response to Shiga Toxin-Producing *Escherichia coli*-Associated Hemolytic–Uremic Syndrome

**DOI:** 10.3390/microorganisms7070195

**Published:** 2019-07-08

**Authors:** Silvia Y. Bando, Priscila Iamashita, Filipi N. Silva, Luciano da F. Costa, Cecilia M. Abe, Fernanda B. Bertonha, Beatriz E. C. Guth, André Fujita, Carlos A. Moreira-Filho

**Affiliations:** 1 Department of Pediatrics, Faculdade de Medicina da Universidade de São Paulo, São Paulo, SP 05403-000, Brazil; 2 Instituto de Física de São Carlos, Universidade de São Paulo, São Carlos, SP 13566-590, Brazil; 3 Laboratory of Bacteriology, Butantan Institute, São Paulo, SP 05503-900, Brazil; 4 Department of Microbiology, Immunology and Parasitology, Universidade Federal de São Paulo, Escola Paulista de Medicina, São Paulo, SP 04023-062, Brazil; 5 Department of Computer Science, Instituto de Matemática e Estatística, Universidade de São Paulo, São Paulo, SP 05508-090, Brazil

**Keywords:** shiga toxin-producing *Escherichia coli*, hemolytic–uremic syndrome, caco-2 cells, enterocyte-bacteria interaction, systems biology, gene co-expression network, weighted gene co-expression network analysis (WGCNA)

## Abstract

Shiga toxin-producing *Escherichia coli* (STEC) O113:H21 strains are associated with human diarrhea and some strains may cause hemolytic–uremic syndrome (HUS). In Brazil, these strains are commonly found in cattle but, so far, were not isolated from HUS patients. Here, a system biology approach was used to investigate the differential transcriptomic and phenotypic responses of enterocyte-like Caco-2 cells to two STEC O113:H21 strains with similar virulence factor profiles (i.e., expressing *stx2*, *ehxA*, *epeA*, *espA*, *iha*, *saa*, *sab*, and *subA*): EH41 (Caco-2/EH41), isolated from a HUS patient in Australia, and Ec472/01 (Caco-2/Ec472), isolated from bovine feces in Brazil, during a 3 h period of bacteria–enterocyte interaction. Gene co-expression network analysis for Caco-2/EH41 revealed a quite abrupt pattern of topological variation along 3 h of enterocyte–bacteria interaction when compared with networks obtained for Caco-2/Ec472. Transcriptional module characterization revealed that EH41 induces inflammatory and apoptotic responses in Caco-2 cells just after the first hour of enterocyte–bacteria interaction, whereas the response to Ec472/01 is associated with cytoskeleton organization at the first hour, followed by the expression of immune response modulators. Scanning electron microscopy showed more intense microvilli destruction in Caco-2 cells exposed to EH41 when compared to those exposed to Ec472/01. Altogether, these results show that EH41 expresses virulence genes, inducing a distinctive host cell response, and is likely associated with severe pathogenicity.

## 1. Introduction

Hemolytic-uremic syndrome (HUS) is a thrombotic microangiopathy clinically defined by thrombocytopenia, non-immune hemolytic anemia, and renal failure. Typical HUS develops secondarily to gastrointestinal infection with Shiga toxin (Stx)-producing *Escherichia coli* (STEC) [1]. The pathogenesis of STEC in intestinal illness usually entails bacterial attachment to the intestinal epithelial cells and microvillus destruction and translocation of Stx [2,3,4,5]. Some STEC strains attach to the enterocytes via the intimin adherence protein, encoded by the *eae* gene that resides on the locus of enterocyte effacement (LEE) pathogenicity island. There are, however, LEE-negative STEC strains, such as O113:H21, that do not produce intimin but can also cause HUS [2,3,4,5]. This serotype harbors several virulence genes, such as *sab*, *subAB*, *ehxA*, and possibly other yet unknown virulence factors, but it is not clear how all these genes/factors act together with Stx in promoting pathogenesis [6,7].

Interestingly, in Brazil, O113:H21 strains are commonly found in cattle but, so far, were not isolated from HUS patients [8,9]. Several studies have been done on STEC strains isolated from animal and environmental sources aiming at identifying virulence genes and characterizing the pathogenic potential of these strains. However, there is no specific virulence genetic profile that enables one to distinguish between the pathogenic and environmental strains [7,8,9]. Thus, the pathogenic potential of the environmental strains remains unknown. Moreover, the molecular mechanisms underlying the capacity of some STEC strains to cause severe disease and the host cell response to HUS-causing strains are not yet completely understood.

In a previous study [7], we were able to show genomic and phenotypic differences between two O113:H21 STEC strains: EH41, isolated from a HUS patient in Australia; and Ec472/01, isolated from bovine feces in Brazil. We also identified potential molecular markers—probably involved in pathogenicity—that were absent in several environmental strains [7]. These results indicated that STEC strains isolated from animal reservoirs often do not have the complete gene repertoire for causing severe diseases, such as HUS.

In the present work, we used a system biology approach to investigate the temporal response of Caco-2 cells to the STEC strains EH41 and Ec472/01 along enterocyte–bacteria interaction. Caco-2 cell line is a widely used model for studying the human intestinal epithelial barrier [10]. The transcriptomic analysis was based on temporal gene co-expression network (GCN) data in order to gain a better understanding on the molecular mechanisms underlying the capacity of the EH41 strain to cause HUS. The phenotypic alterations in Caco-2 along enterocyte–bacteria interaction were assessed by scanning electronic microscopy (SEM).

## 2. Materials and Methods

### 2.1. Bacterial Strains

EH41 and Ec472/01 strains were provided by the collection kept at the Department of Microbiology, Immunology and Parasitology, Escola Paulista de Medicina, UNIFESP, São Paulo, Brazil. EH41 was isolated from a child with HUS in Australia [11], and Ec472/01 was isolated from cattle feces in Brazil [8]. The serotype, cytotoxic activity, virulence factors, and enterohaemolytic phenotype of these strains were previously investigated [8,12]. The *eae*-negative strains EH41 and Ec472/01 express Stx type 2 and harbor many known virulence genes, such as *ehxA, epeA, espA, iha, saa, sab, and subA* [12]. However, Ec472/01 does not harbor the potential virulence factors *dicA*, *dicC*, *fecC*, *Ecs1176*, *insA*, and *rusA*, which are all present in EH41 [7]. In all biological and molecular assays described in the next sections, a single colony of each strain was inoculated in trypticase soy broth (TSB) and grown at 37 °C for 18 h.

### 2.2. Cell Culture Procedures and Temporal Shiga toxin (Stx)-producing Escherichia coli (STEC)-Enterocyte Interaction Assays

The Caco-2 cells (ATCC^®^ HTB-37™) were cultured in T flasks (25 cm^2^) or in 4-well cell culture plates containing glass coverslips (13 mm) and Dulbecco’s modified eagle’s medium (DMEM) with 10% fetal bovine serum (FBS) and penicillin–streptomycin (100 U/mL-100 μg/mL) in a 5% CO_2_ at 37 °C. The cells were grown until confluence and the formation of a polarized epithelial cell monolayer (5–7 days). Twenty-four hours prior to interaction assays, the cells were washed three times with 1× phosphate buffered saline (PBS) and incubated with antibiotic-free DMEM containing 10% FBS. Interaction assays were performed with 400 µL or 50 µL (Abs_550_ = 0.35) of TSB grown bacterial culture, respectively placed on a Caco-2 monolayer cultured in T flasks or in 4-well cell culture plates (around 100 bacteria per human cell). Subsequently, the cell cultures were incubated in 5% CO_2_ at 37 °C for different time intervals. After each time interval, Caco-2 cells were recovered for RNA extraction or for SEM procedures (see below). The time series—24 sequential intervals of 7.5 min each—encompassed a total of 25 samples (T0 to T24). In T0, the Caco-2 cells were recovered just after bacterial inoculation.

### 2.3. RNA Extraction

After enterocyte–STEC interaction, the cells were washed five times with 1× phosphate buffered saline (PBS). Subsequently, Caco-2 cells were lysed directly in the culture flasks with 600 µL of Buffer RLT (Qiagen, Valencia, CA, USA). The cell lysate was collected and total RNA extraction was accomplished using an RNeasy Mini kit (Qiagen, Valencia, CA, USA). RNA purity analysis and quantification were done using a NanoVue spectrophotometer (GE Healthcare Life Sciences, Marlborough, MA, USA). RNA quality was assessed on an Agilent BioAnalyzer 2100 (Agilent, Santa Clara, CA, USA). All samples presenting RIN ≥7.0 were stored at −80 °C until used in hybridization experiments.

### 2.4. Microarray Hybridization

In order to determine gene expression profiles, 4 × 44K v.2 DNA microarrrays (whole human genome microarray kit, Agilent technologies, cat no. G4845A, Santa Clara, CA, USA) were used. The procedures for hybridization using the fluorescent dye Cy3 followed the manufacturer’s protocols (one-color microarray-based gene expression analysis—low input quick amp labeling). The images were captured by the reader Agilent bundle, according to the parameters recommended for bio-arrays and extracted by Agilent feature extraction software version 9.5.3. Spots with two or more flags (low intensity, saturation, controls, etc.) were considered as NA, that is, without valid expression value. An in-house algorithm in R environment (version 3.4.4 [13]) was used for excluding transcript spots presenting one or more NAs and for converting gene expression values to log base 2. Through this procedure, we identified 6876 or 7555 valid gene ontology (GO) annotated genes for Caco-2/EH41 or Caco-2/Ec472/01 groups, respectively. Data normalization was performed using limma package [14] in R environment (version 3.4.4 [13]). All microarray raw data have been deposited in expression omnibus (GEO) public database under accession number GSE104492.

### 2.5. Systems Biology Approach

**Figure 1:**
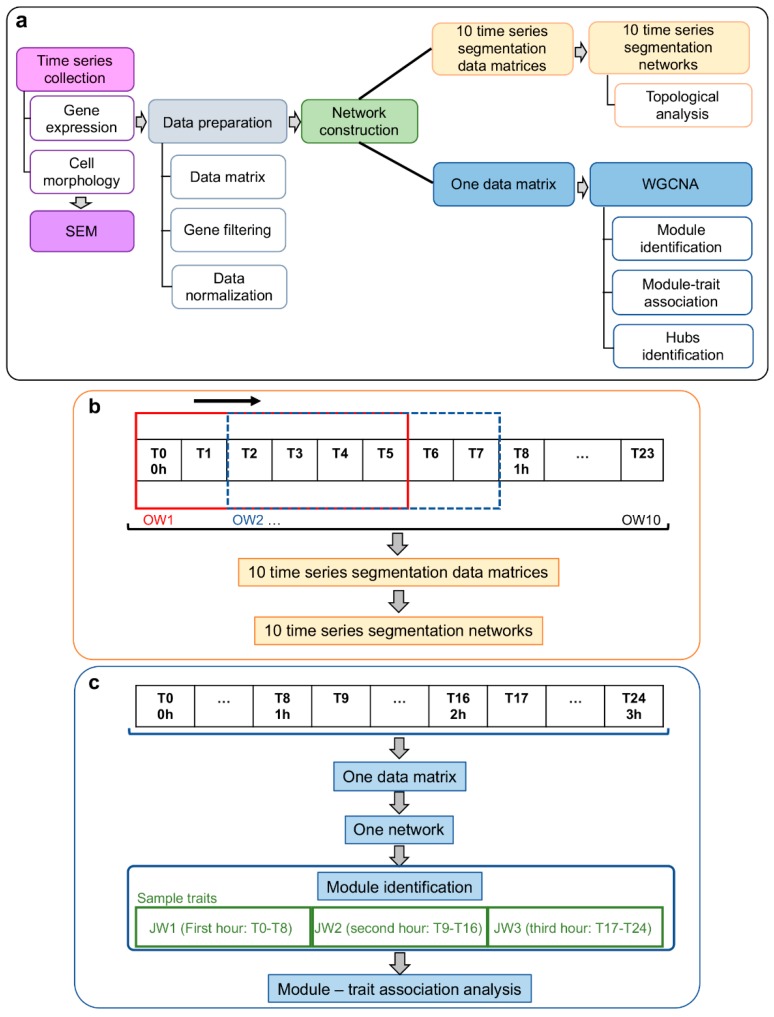
Workflow of time series collection and analysis for investigating Caco-2 cells-STEC interactions. (**a**) Time series data analysis and the 3D network and WGCNA approaches for network construction. (**b**) Sliding-window analysis for investigating temporal dynamic network variations along three hours of enterocyte–bacteria interaction. Each overlap window (OW) represents a fifteen minutes interval and each window included data from six sequential time intervals, i.e., the red box indicates the first window (OW1) and the dashed blue box indicates the second window (OW2). (**c**) Gene network construction by WGCNA for investigating module–trait associations. STEC, Shiga toxin (Stx)-producing *Escherichia coli*; WGCNA, Weighted Gene Co-Expression Network Analysis; JW, juxtaposed windows.

A sliding-window analysis was performed to investigate temporal gene co-expression network (GCN) variation along 3 h of enterocyte–STEC interaction. This time-series adopted equal intervals of 7.5 min during 3 h. We used two approaches for GCN construction: (i) networks based on differentially expressed GO annotated genes only for topological analysis, and (ii) networks encompassing all GO annotated genes for module analysis associated with three intervals. Temporal phenotypic variation was assessed through scanning electron microscopy (SEM). The experimental approach workflow is depicted in Figure 1.

### 2.6. Gene Co-Expression Network (GCN) Topological Analysis

The normalized time series gene expression data for Caco-2 exposed to EH41 (Caco-2/EH41) or to Ec472/01 (Caco-2/Ec472) were grouped in 10 overlapping windows (OW). Each overlap (sliding window) represents a fifteen minutes interval and each window included data from six sequential time intervals, as follows: the first window (OW1) encompassed T0 until T5 intervals; the OW2 encompassed T2 to T7; and successively, until OW10 (T18 to T23) (Figure 1b). Differentially expressed transcripts for Caco-2/EH41 and for Caco-2/Ec472 were obtained using analysis of variance (ANOVA) and adjusted Bonferroni correction. Gene co-expression networks for DE GO annotated genes were constructed for Caco-2/EH41 and for Caco-2/Ec472 groups based on Pearson’s correlation (*r* coefficient). The GCN correlation thresholds were chosen in order to ensure that most nodes continued to be connected to the network major components (i.e., major transcriptional modules) and that the network remained stable (based on network connectivity and modularity measures) along a threshold range, i.e., maintaining network’s topological structure [15]. We adopted an *r* coefficient cut-off |*r*| ≥ 0.984 for Caco-2/EH41 group and |*r*| ≥ 0.990 for Caco-2/Ec472 group after testing for a threshold range between 0.700 ≤ |*r*| ≤ 0.999. This test was performed for OW1 networks.

Network visualization was accomplished by using a force directed algorithm in which connected nodes are brought close together while disconnected nodes are kept as far as possible from each other. A more detailed description of this method appears in Bando et al. [15]. In particular, we adopted the software Networks 3D—developed by ourselves—which allows the visualization of large complex networks [16]. We also employed a node degree distribution analysis to track networks’ topological changes [16].

### 2.7. Weighted Gene Co-Expression Network Analysis (WGCNA): Module–Trait Association

The normalized time series data of all GO annotated genes (6876 genes for Caco-2/EH41 or 7555 for Caco-2/ Ec472) of the 25 time-interval samples were used for WGCNA (Figure 1c). Networks were constructed using the WGCNA package [17] in R environment (version 3.4.4 [13]). Pearson’s correlation coefficient was used for obtaining gene co-expression similarity measures and for the subsequent construction of an adjacency matrix using soft power and topological overlap matrix (TOM). Soft-thresholding process transforms the correlation matrix to mimic the scale-free topology. TOM is used to filter weak connections during network construction. Module identification is based on TOM and in average linkage hierarchical clustering. Keeping to the scale-free topology criterion, soft power *β* = 16 was considered for both networks (Figure S1). Finally, dynamic cut-tree algorithm was used for dendrogram’s branch selection. The module eigengene is defined as the first principal component of a given module, which can be considered a representative of the gene expression profiles in a module [17].

#### 2.7.1. Module–Trait Association

The 25 time-interval samples were grouped in three juxtaposed windows (JW) with the first nine sequential time intervals grouped in the initial window (JW1, first hour of bacterial exposure) and eight sequential intervals grouped in each of two other windows (JW2; JW3, second and third hour of bacterial exposure, respectively) (Figure 1c). Consequently, we obtained the gene significance (GS), which is a value of the correlation between the trait (here is represented by JW) and the gene expression values [17]. The mean GS for a particular module is considered as a measure of module significance (MS). The GS values were obtained using Pearson’s correlation and to assign a *p*-value to the module significance, we used Student’s *t* test. The modules, which presented high correlation value |*r*| ≥ 0.5 and *p* < 0.01, were selected for biological functional analysis of module genes.

#### 2.7.2. Functional Enrichment Analysis of Network Module Genes

KEGG pathway enrichment analysis was performed using the Enrichr online web-based tool [18,19] for the WGCNA modules that are associated with the windows JW1, JW2, and JW3 (*p* < 0.05).

#### 2.7.3. Intramodular Analysis for Hub Selection

Intramodular node connectivity was calculated considering: (i) kTotal, the whole network connectivity of each gene; (ii) kWithin, gene connections with other genes in the same module [17]. We plotted all gene values in a kTotal vs kWithin graphic for gene categorization. Genes presenting high kTotal and kWithin were considered as hubs in the module.

### 2.8. Scanning Electron Microscopy (SEM)

After Caco-2-STEC interaction assays, the cells exposed to bacteria were gently washed three times with 1 × PBS and fixed with Karnovsky fixative solution for at least 24 h at 4 °C. After fixation, cells were washed three times with 0.1 M cacodylate buffer (10 min) and post- fixed with 1% osmium tetroxide (prepared in the same buffer) for 30 min. After being washed for three times with distilled water, preparations were dehydrated through a graded ethanol series (50%, 75%, 85%, 95%, and 100%). Subsequently, the preparations were dried (critical point method), mounted on stubs, and sputter coated with gold. Specimens were then examined under SEM (QUANTA 250, FEI Company, Eindhoven, Netherlands) at 12.5 kV.

### 2.9. Validation of Microarray Data by RT-qPCR

Differential gene expression data were validated through quantitative real-time polymerase chain reaction (qPCR). Specific primers for selected genes (Table S1) were designed using the Primer-BLAST (Primer3 Input, version 0.4.0 and BLAST, available at: http://www.ncbi.nlm.nih.gov/tools/primer-blast/). All samples were amplified in triplicates. Amplification reactions were performed in a 25 μL final volume containing 1× SYBR Green mix (Quantitec SYBR Green PCR kit, QIAGEN, Hilden, Germany), 10 pmol of each primer, and 2 µL cDNA (1/10 dilution, synthesized from 1 µg of total RNA). Real time PCR amplifications were performed in applied biosystems stepone plus real time PCR System with StepOne software (Applied biosystems, Foster City, CA, USA) with the following cycling parameters: an initial hot start of 95 °C for 15 min followed by 40 cycles of 95 °C for 15 s and 60 °C for 30 s. In order to normalize qPCR reactions, two genes—*RPLP0* for Caco-2/EH41 and *MRPL19* for Caco-2/Ec472—were used as reference genes after checking that amplification curves for four RNA samples (corresponding to four different time intervals) of each window yielded essentially the same results. Relative expression was determined by the relative standard curve method [20].

## 3. Results

### 3.1. Transcriptional and Network Topology Analysis

**Figure 2:**
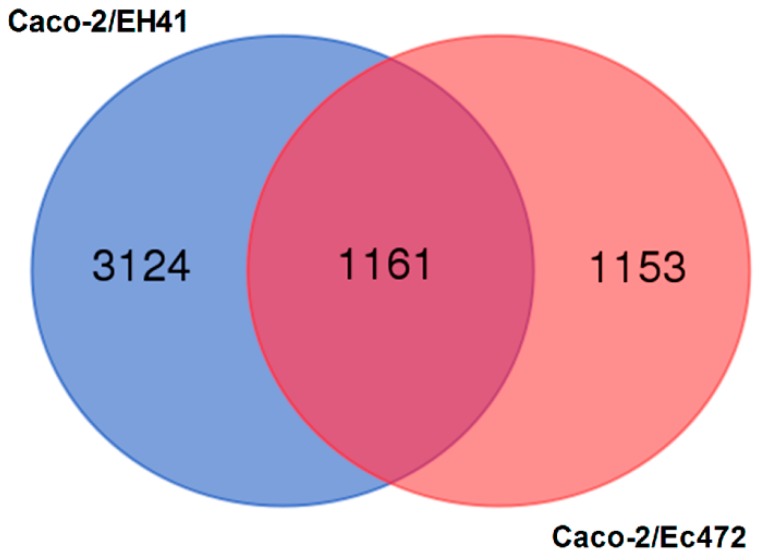
Differential gene expression analysis of Caco-2 cells during three hours of enterocyte–STEC interaction. Venn diagram for differentially expressed (DE) genes in Caco-2/EH41 and Caco-2/Ec472 groups.

**Figure 3:**
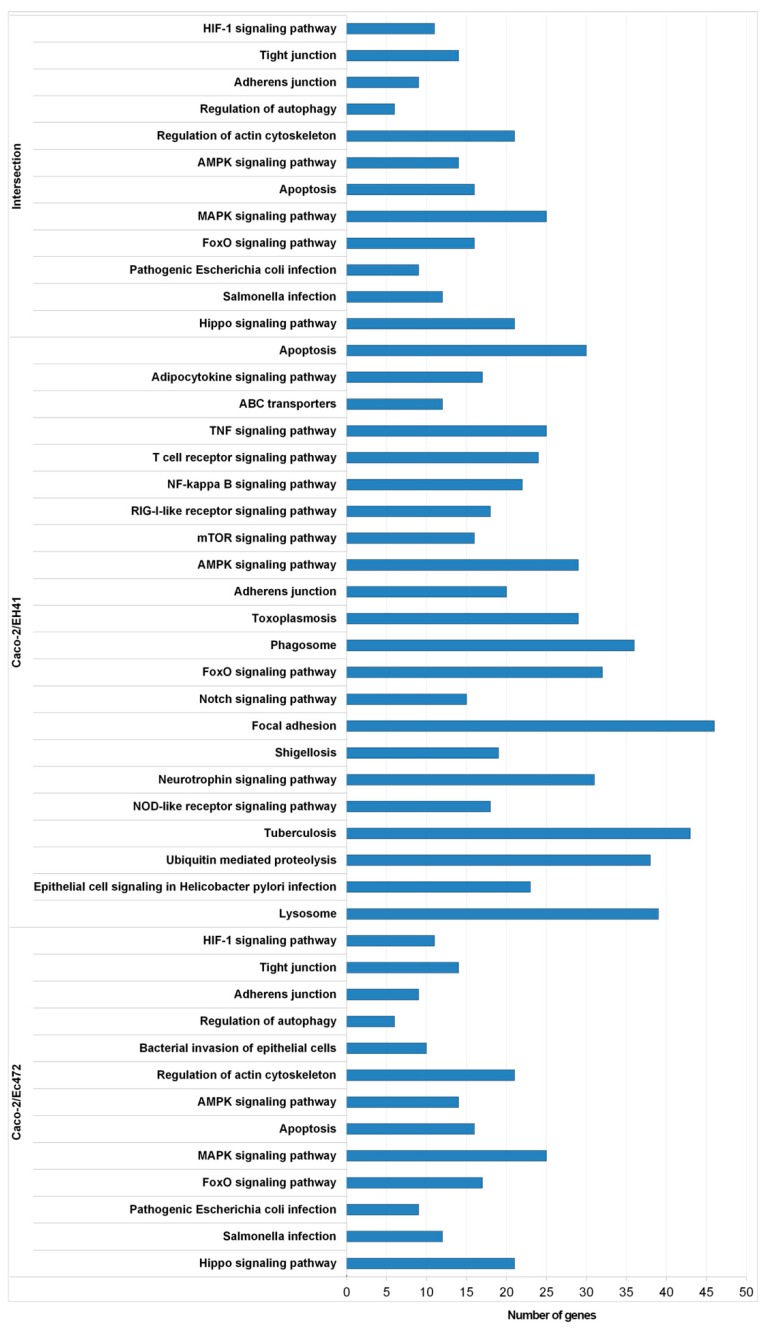
DE genes enrichment analysis based on KEGG pathways. Distribution of the KEGG pathways found in three sets of DE genes. Intersection stands for common DE genes in Caco-2/EH41 and in Caco-2/Ec472 groups. Caco-2/EH41 stands for DE genes present only in Caco-2/EH41 group. Caco-2/Ec472 stands for DE genes present only in Caco-2/Ec472 group.

The initial analysis aimed to study the global gene expression variation (fifteen minutes intervals) of Caco-2 cells during 3 h of interaction with EH41 (Caco-2/EH41 group) or with Ec472/01 (Caco-2/Ec472 group). The comparative analysis of the 10 windows (OW1–OW10) was performed for each group separately (see Materials and Methods and Figure 1b). We obtained 4285 or 2314 differentially expressed (DE) genes corresponding, respectively, to Caco-2/EH41 or to Caco-2/Ec472 groups (using ANOVA and adjusted Bonferroni correction). All the DE genes were analyzed through Venn diagram (Figure 2). The result showed that 3124 out of 4285 DE genes are DE only in the Caco-2/EH41 group, whereas 1153 out of 2314 DE genes are DE only in the Caco-2/Ec472 group. The remaining 1161 DE genes are common for both groups. This analysis shows that EH41 induces a greater gene expression dysregulation in in Caco-2 cells. KEGG pathway-based enrichment analysis for these three DE gene sets showed that in the Caco-2/EH41 group, a total of 317 DE genes are over-represented in pathways related to inflammation, phagocytosis, and adaptive immune response, while only 164 DE genes appear in these pathways in the Caco-2/Ec472 group. Interestingly, 117 of the DE genes common to both groups are over-represented in the pathways related to cellular cytoskeleton, cell–cell junction, and innate immune response (Figure 3). The complete list of the genes over-represented in KEGG pathways for the three sets of DE genes (*p* < 0.05) are presented in Table S2.

**Figure 4:**
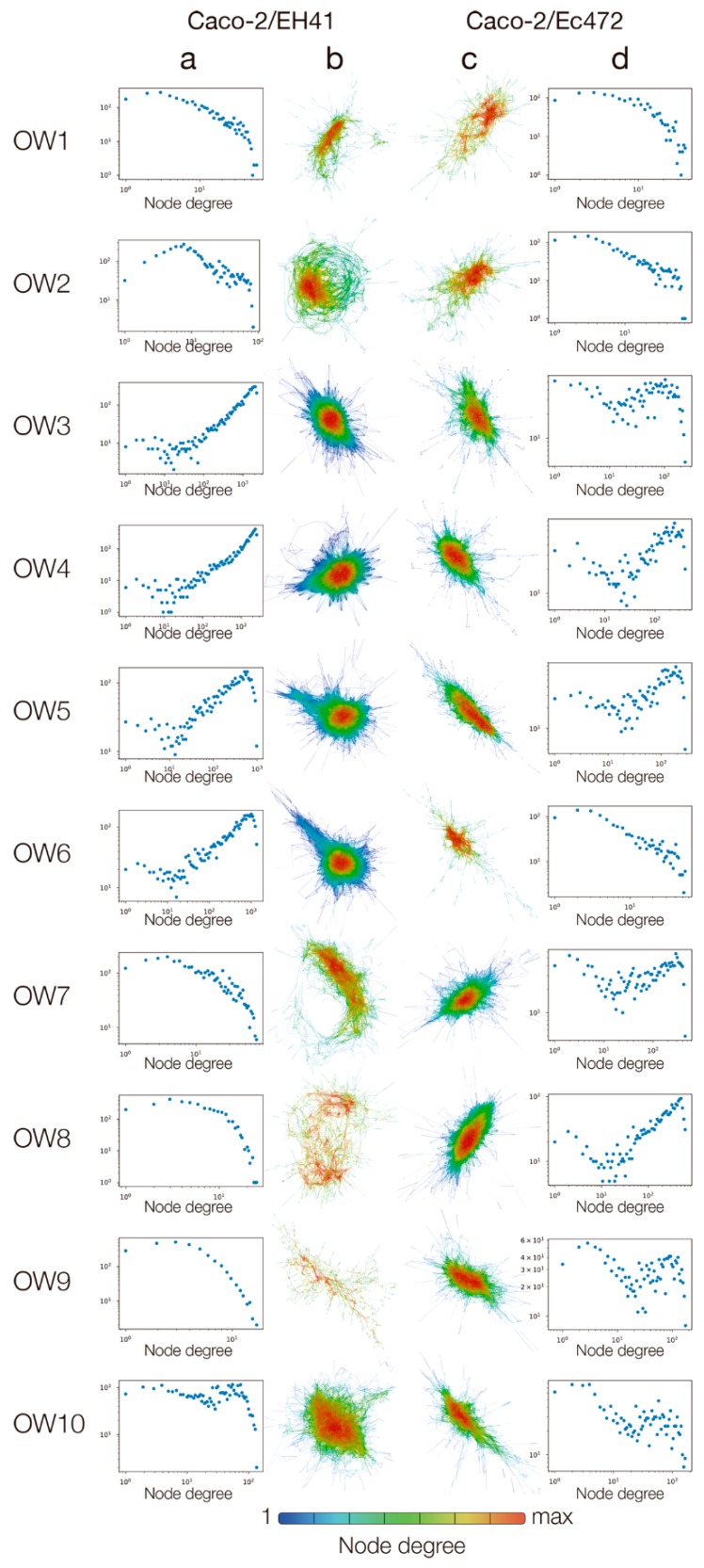
Dynamic DE networks of Caco-2 cells along three hours of enterocyte–STEC interaction. Node degree distributions and gene co-expression networks for Caco-2/EH41 (**a**,**b**) and Caco-2/Ec472 (**c**,**d**) groups in 10 overlapping windows (OW1–10). In the Caco-2/EH41 group, anomalous degree distributions appear in OW3–OW6, recovery from this regime occurs in OW7–OW9, and the anomalous status is regained in OW-10. In Caco-2/EH472, only the networks for OW1 and OW2 display a regular degree distribution.

Total DE genes were used for constructing ten GCNs for Caco-2/EH41 and for Caco-2/Ec472 groups. The networks were constructed by Pearson’s correlation and network visualization was accomplished by using a force directed algorithm. Node degree distributions for each respective network are shown in Figure 4. These results allowed us to track the changes of network topological characteristics of Caco-2 cell response along enterocyte–bacteria interaction at fifteen minute intervals (i.e., at overlapping windows). The networks for Caco-2/EH41 group revealed a quite abrupt pattern of topological variation along enterocyte–bacteria interaction (Figure 4a,b) compared with networks for Caco-2/Ec472 group (Figure 4c,d). In the Caco-2/EH41 group, the OW1 and OW2 networks present node degree distributions that are more compatible with those commonly found in real-world networks, i.e., decreasing frequency of nodes with their number of connections. However, a strikingly different behavior was found for the following three sequential networks (OW3–OW5), which presents anomalous degree distributions, such as having more highly connected nodes than nodes with lower degrees. It is interesting to note that the networks in the OW7–OW9 interval seem to recover from this anomalous regime, whereas the OW10 network regains this status again. Conversely, Caco-2/Ec472 networks showed a relatively smooth pattern of topological variation along enterocyte–bacteria interaction. In this group, only the networks for the windows OW1 and OW2 display a regular behavior; all the other sequential networks present anomalous degree distributions.

### 3.2. Weighted Gene Co-Expression Network Analysis (WGCNA)

**Figure 5:**
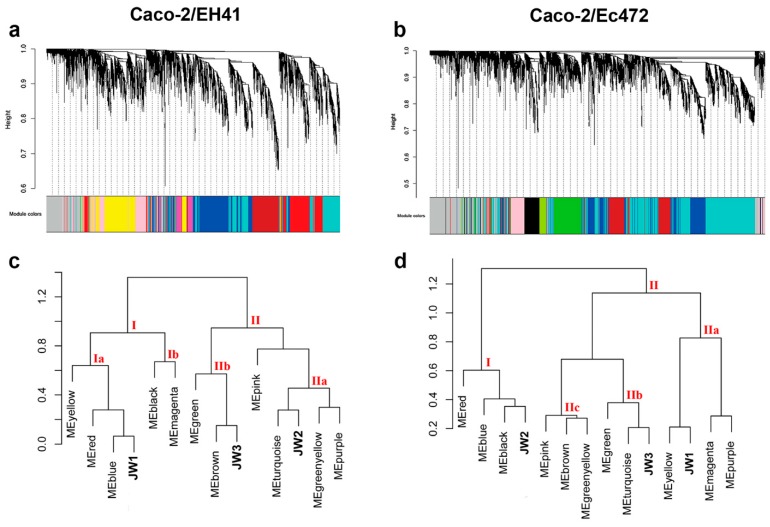
WGCNA for Caco-2/EH41 and Caco-2/Ec472 groups. Hierarchical clustering dendrogram and module identification, indicated by different colors, for Caco-2/EH41 (**a**) and Caco-2/Ec472 (**b**). Eigengene module dendrogram obtained for Caco-2/EH41 (**c**) and for Caco-2/Ec472 (**d**). Red roman numbers indicate the major meta-modules.

Two global gene expression networks were constructed for WGCNA (Figure 1): one for Caco-2 exposed to EH41 (Caco-2/EH41 group), and another for Caco-2 exposed to Ec472/01 (Caco-2/Ec472 group). Hierarchical clustering dendrogram identified 11 distinct gene modules for each group (Figure 5a,b). Module size ranged from 112 (green–yellow module) to 1010 (turquoise module) genes for the Caco-2/EH41 group, and from 118 (green–yellow module) to 2472 (turquoise module) genes for the Caco-2/Ec472 group.

#### 3.2.1. Caco-2/EH41 Network

The Caco-2/EH41 eigengene module dendrogram presented two major meta-modules, here designated I and II (Figure 5c). It is interesting to note that the meta-module I encompasses the window JW1 and this window is closely related with the blue module. The meta-module II encompasses two sub-meta-modules, IIa and IIb, that are related with the windows JW2 and with JW3, respectively. Moreover, the modules turquoise and brown are closely associated with the windows JW2 and JW3, respectively.

**Figure 6:**
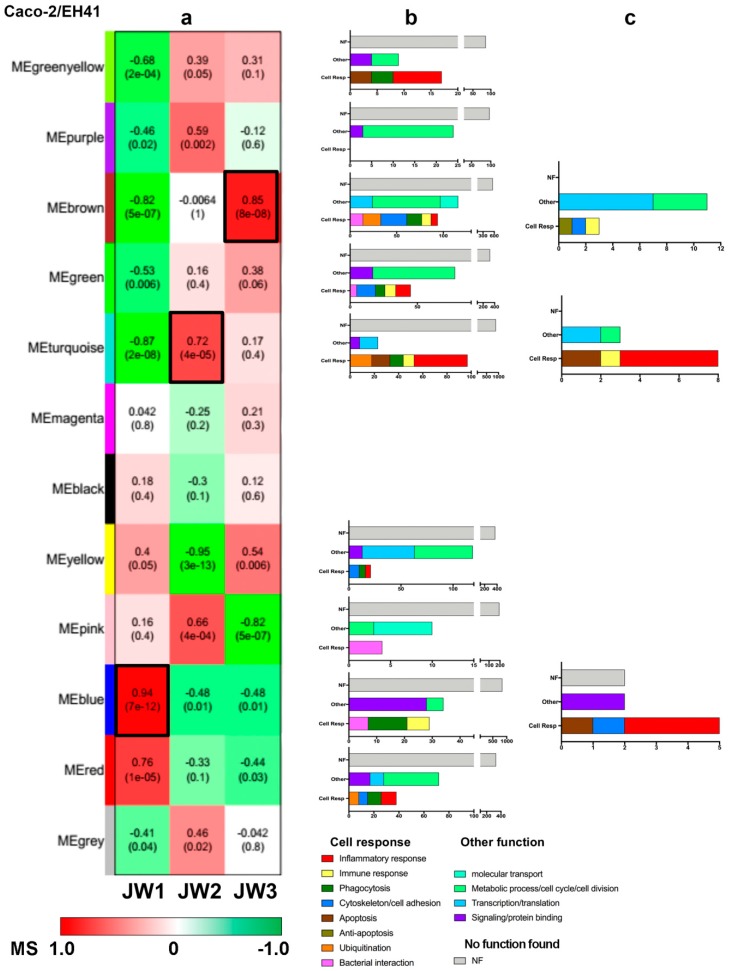
Module characterization of the Caco-2/EH41 group. (**a**) Heatmap of the relationship between modules and the three temporal windows (JW1, JW2, and JW3). Color bar indicates the module significance (MS) value ranged from 1 to −1. Numbers in the heatmap indicate a module correlation value and the numbers in parenthesis indicate *p*-values. The blue, turquoise, and black modules presented the highest association with JW1, JW2, and JW3, respectively. These modules are indicated by black borders. (**b**) KEGG enrichment analysis of the nine modules significantly associated with at least one window. (**c**) Functional profile of the hubs contained in the blue, turquoise, and black modules.

Module–window correlation analysis identified nine modules—green–yellow, purple, brown, green, turquoise, yellow, pink, blue, and red—that were significantly (*p* < 0.05) associated with at least one window (Figure 6a). The KEGG enrichment analysis showed that all modules, except the purple module, encompassed many genes involved in cell responses to bacteria, such as inflammatory response and phagocytosis (Figure 6b and Table S3). It is worth mentioning that the majority of the genes involved in the inflammatory response are found in the modules green–yellow and turquoise, which are negatively and positively correlated with windows JW1 and JW2, respectively.

Since the blue, turquoise, and brown modules presented the highest and positive correlations values with windows JW1, JW2, and JW3, respectively (Figure 6a), we categorized the genes of these modules through intramodular connectivity measures in order to identify the hubs, i.e., the network nodes presenting high kTotal and kWithin (Figure S2). Table 1 presents the biological function of these hubs. In JW1 and JW2, several hubs are involved in innate immune response, where inflammation seems to be the more important cell response to EH41 (Figure 6c).

The blue module, correlated with JW1 (all hubs are hyper-expressed in JW1, see Table S4), has three out of nine hub genes involved in inflammatory response. One of these hubs is *ADRA1A*, which positively regulates ERK1/2 and MAPK cascades. It has been described that Stx can induce these kinase cascades and ERK1/2 activation in intestinal epithelial cells in vitro, and in the colon of infant rabbits, subsequently causing intestinal inflammation [21]. Another hub, *TBKBP1*, encodes for an adaptor protein that binds to TBK1 and is involved in the TNF/NFKB cascade [22]. Moreover, TBK1 is essential for autophagic process [23]. The third hub, *CSH1*, encodes for a protein involved in JAK-STAT cascade [24]. It is interesting to mention that enterohemorrhagic *Escherichia coli* inhibits the activation of STAT-1 and then modulates the host inflammatory response [25]. The third hub, *MEG3*, is possibly related with cell response to bacteria, since it encodes for a long noncoding RNA whose overexpression induces apoptosis in enterocytes [26].

The turquoise module, associated with JW2 (nine hubs are hyper-expressed and two—*UFC1* and *YY1AP1*—are hypo-expressed in JW2, Table S4), has eight out of its 11 hubs involved in the immune response. It should be noted that five of those hubs are related to the inflammatory response, what seems to be the most important biological process along this time interval. Indeed, four of these hubs—*TICAM1* (alias *TRIF*), *BCL3*, *TNFRSF1A*, and *BIRC3*—codify for proteins involved in I-kappa B kinase regulation and NF-kappa B signaling [27,28,29]. Moreover, *TRIF* is involved in host–pathogen communication via TLR5 in the intestinal epithelium [30]. The fifth hub, *IRF1*, encodes for protein involved in pro-inflammatory process. IRF1 induces IL-7 production that is essential for the regulation of the immune response in the human intestinal mucosa [31]. Additionally, in this module there are two other hubs, *BBC3* and *UFC1*, which are related to apoptotic process. It is interesting to note that *BBC3* is involved in intestinal epithelial cell apoptosis and contributes to ulcerative colitis in mice [32]. On the other hand, Yu et al. [33] showed that *UFC1* promotes the cellular proliferation while attenuating anti-apoptosis in colorectal cancer. The last hub in this module, *DOK1*, encodes for an inhibitor of signaling precursors, RAS/ERK and PI3K/Akt, and acts in immune response modulation [34].

The brown module, associated with JW3 (all hubs are hyper-expressed in JW3, Table S4), has five out of its 16 hubs*—NUP62*, *MARCKS, HIST1H2BJ, UBE2Q1*, and *PARL*—involved in cell response to bacteria. The hubs *NUP62* and *PARL* are involved in the anti-apoptotic process [35,36]. Another hub, *MARCKS*, encodes a protein involved in actin cytoskeleton regulation [37]. Moreover, this gene may be involved in the expression of pro-inflammatory cytokines in macrophages after induction by LPS [38]. *HIST1H2BJ* is involved in innate immune response [39,40] and *UBE2Q1* encodes for a protein involved in ubiquitination.

#### 3.2.2. Caco-2/Ec472 Network

The Caco-2/Ec472 eigengene module dendrogram has two major meta-modules, I and II (Figure 5d). The meta-module I includes three modules, and one of them, the black module, is closely related with JW2. The meta-module II encompasses the majority of the gene modules (eight out of 11), and it can be subdivided in three meta-modules: IIa, IIb, and IIc. One of these meta-modules, IIa, is related to the window JW1 and closely associated with the yellow module. The other meta-module, IIb, is related with the window JW3 and closely associated with the turquoise module.

**Figure 7:**
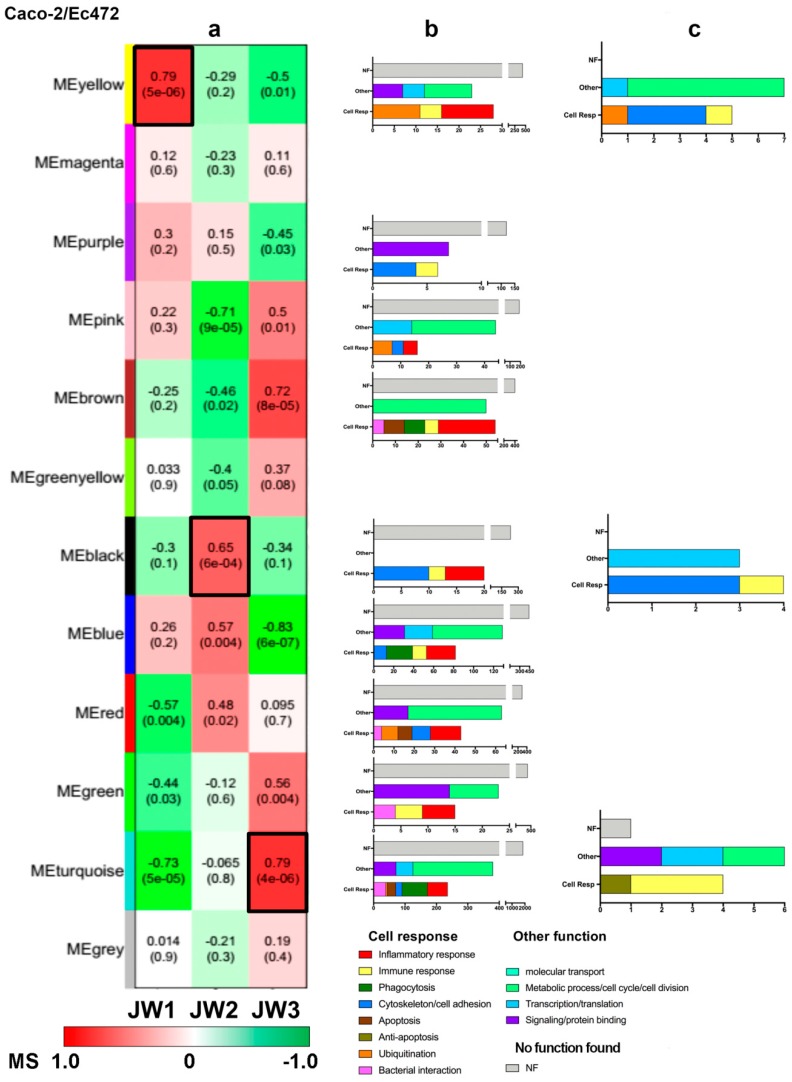
Module characterization of the Caco-2/Ec472 group. (**a**) Heatmap of the relationship between modules and the three temporal windows (JW1, JW2, and JW3). Color bar indicates the module significance (MS) value ranged from 1 to −1. Numbers in the heatmap indicate a module correlation value and the numbers in parenthesis indicate *p*-values. The yellow, black, and turquoise modules presented the highest association with JW1, JW2, and JW3, respectively. These modules are indicated by black borders. (**b**) KEGG enrichment analysis of the nine modules significantly associated with at least one window. (**c**) Functional profile of the hubs contained in the yellow, black, and turquoise modules.

In the Caco-2/Ec472 network, we identified nine modules—green-yellow, purple, brown, green, turquoise, yellow, pink, blue, and red—that were significantly (*p* < 0.05) associated with at least one window (Figure 7a). The KEGG enrichment analysis showed that all modules possess several genes involved in the cellular responses to Ec472/01 such as inflammatory response, phagocytosis, and apoptosis (Figure 7b, Table S5).

In order to identify hub genes, we selected the yellow, black, and turquoise modules because these modules presented the highest positively correlated values with JW1, JW2, and JW3, respectively (Figure S2). The biological functions of these hubs are listed in Table 2. Different to what was found for the Caco-2/EH41 group, where many hubs are related to inflammatory response, in this group, the hub-containing modules encompass genes mostly involved in cytoskeleton organization and cell–cell adhesion (Figure 7c).

The yellow module, associated with JW1 (all hubs are hyper-expressed in JW1, Table S6), has four out of its 12 hubs involved in cell response to bacteria. Three hubs, *CCNA2*, *EMP2*, and *PODXL*, are involved in cytoskeleton-related functions and another hub, *KCNN4* (alias *KCa3.1*), is involved in immune response and could be related to inflammation [41,42]. *KCNN4* encodes for a protein of calcium-activated potassium channel involved in the migration of human epithelial cells. It was previously shown that abnormal epithelial cell migration could contribute to intestinal inflammation [42]. It is interesting to note that three hubs in this module—*CHAF1A, RAD51*, and *UBE2T*—are involved in DNA repair [43,44,45]. Bielaszewska et al. [46] showed that some bacterial toxins, such as CdtV-B, expressed by enterohemorrhagic *E. coli* O157, cause DNA damage and activate DNA repair mechanisms.

The black module, associated with JW2 (all hubs are hyper-expressed in JW2, Table S6), has three hubs—*RAC2*, *KRT9*, and *KRTAP10-10*—coding for proteins related to cytoskeleton. *RAC2* is also involved in a signaling pathway associated with inflammatory response [47] and *ITGA9* encodes for a binding protein involved in cell adhesion and it is involved in PI3K-Akt signaling pathway.

In the turquoise module, associated with JW3 (nine hubs are hyper-expressed and two, *DOK4* and *NOTCH2NL*, are hypo-expressed in JW3, Table S6), five out of 11 hubs are related to immune response [48,49,50,51,52]. Three of them, *UPF1*, *HAX1*, and *TRAFD1*, code for proteins acting on immune response modulation. The protein UPF1 acts with Regnase-1, and it could be LPS-induced, restricts inflammation, and maintains immune homeostasis [50]. HAX1 (alias HCLS1) is a multifunctional protein involved in cell protective mechanism, such as reduced DNA fragmentation and decreasing Caspase-9 cleavage [49]. TRAFD1 (alias FLN29) acts as a negative regulator of excessive microbial immune response through Toll-like receptor and retinoic acid-inducible gene I (RIG-I)-like helicase signaling pathway [51].

### 3.3. SEM

**Figure 8:**
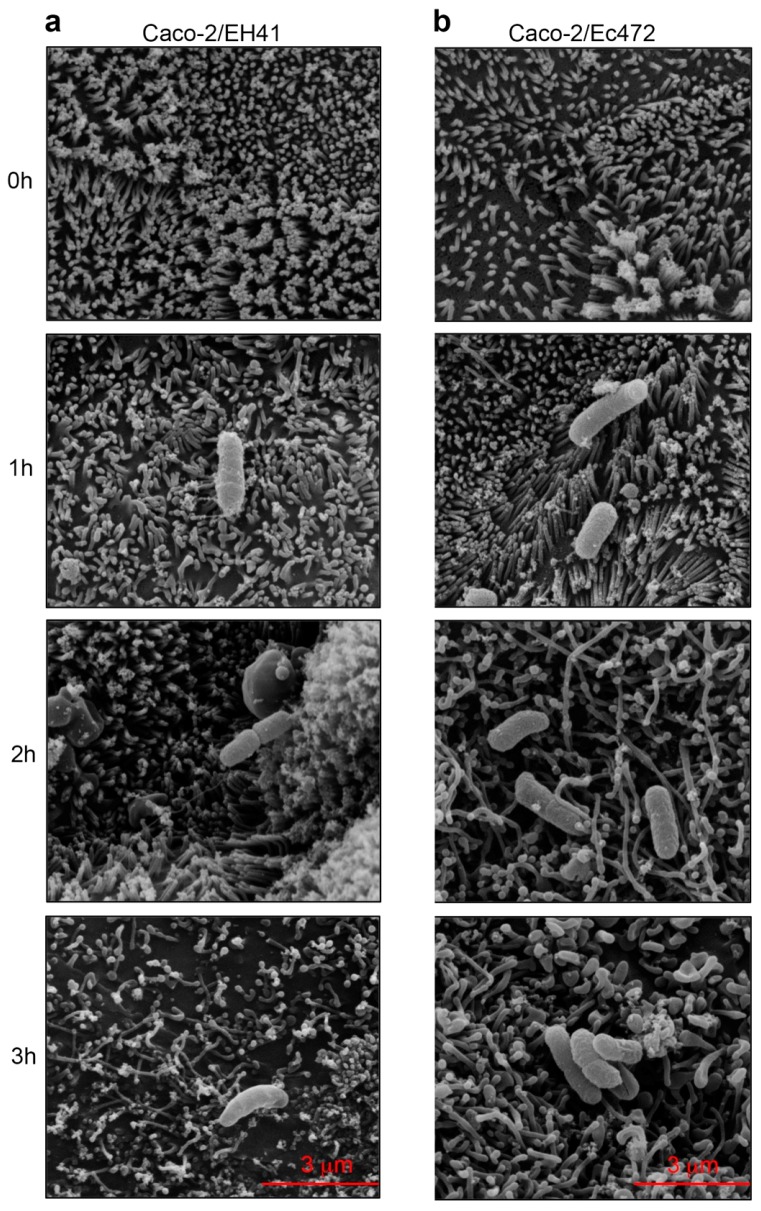
Serial SEM images of Caco-2-STEC interaction. Time series of STEC strains EH41 (**a**) and Ec472 (**b**) interaction with Caco-2 cells (Caco-2/EH41 and Caco-2/Ec472, respectively) at 0 h and three sequential intervals of 1 h each. Bacteria adhered to the cells were observed after 1 h of exposure. In the interval 1–3 h, EH41 caused progressive loss of microvilli. After 1 h, Ec472 also caused morphological changes of the cells, which were characterized by a decrease in microvilli number and their elongation from 2 h to 3 h.

Figure 8 shows the SEM images obtained for Caco-2 along cell–bacteria interaction with strains EH41 or Ec472/01 at four time-intervals (0, 1 h, 2 h, and 3 h). After 1 h, bacteria appear adherent to the cells. In the enterocytes exposed to the EH41 cell, surface alterations, such as progressive loss of microvilli, can be observed. These alterations are visible at 1 h and the process culminates with an extensive loss of microvilli after 3 h of exposure. Here, it is interesting to mention that two hubs in the Caco-2/EH41 network (WGCNA) are related to cytoskeleton: *ROPN1L* (blue module, associated with JW1) encodes for an epithelial cilium structural protein and is involved in its movement [53], and *MARCKS* (brown module, associated with JW3) encodes for a protein of the actin filament component [37]. Moreover, MARCKS may be involved in macrophage pro-inflammatory cytokine expression after LPS induction [38].

Caco-2 cells exposed to Ec472/01 also presented morphological alterations, however, they are distinct in form and in time of occurrence. After 1 h of exposure, loss of microvilli is not yet observable, but the cells present some morphological changes, such as loss of spatial orientation. In the 2–3 h interval, there is a decrease in the number of microvilli and the microvilli become elongated. Noteworthily, in the Caco-2/Ec472 network (WGCNA) there are more hubs, when compared with the Caco-2/EH41 network, involved in cytoskeleton structure or organization. In fact, three hubs associated with JW1 (yellow module) are related to cytoskeleton: *PODXL* encodes for actin cytoskeletal protein [54] and the other two hubs, *EMP2* and *CCNA2*, are involved in cytoskeleton organization and in the regulation of actin reorganization, respectively [55,56]. The turquoise module (JW3) encompasses two hubs, *KRT9* and *RAC2*, which codify for a structural constituent of cytoskeleton and for a GTPase involved in actin filament organization, respectively [47].

### 3.4. RT-qPCR Technical Validation

In order to validate the gene expression patterns identified in microarrays data, the transcript levels of some genes characterized by higher expression were checked by RT-qPCR. Expression profiles of three genes that presented highest GS values were selected for the Caco-2/EH41 group (*TBKBP1, PRIMA1, NEGR1*, all from the blue module, JW1 group) and for the Caco-2/Ec472/01 group (*TNFRSF1A, SLC25A25, BCL7B* all from the blue module, JW3 group). The RT-qPCR results were consistent with the results of the microarray analysis (Figures S3 and S4).

## 4. Discussion

In this study, we investigated the temporal dynamics of the Caco-2 cells responses to STEC EH41 and Ec472/01 strains. Grouping time series data through sliding window methodology is a well-known strategy for investigating point time changes [57,58]. Firstly, we analyzed the gene expression variation at fifteen minutes intervals along three hours of bacteria–enterocyte interaction for the Caco-2/EH41 and Caco-2/Ec472 groups. We found two times more DE genes in the Caco-2 cells exposed to EH41.

Subsequently, we analyzed GCN topological variation along the three hours of bacteria–enterocyte interaction for each STEC strain. Network topology is directly related to node degree distribution: real-world networks, such as GCN and protein–protein networks, have node degree distributions following a power law (scale-free networks) [59]. Gene expression perturbations, such as those involved in STEC-enterocyte interaction [7], alter gene–gene interactions and, consequently, modify network topology. Many studies have shown that topological alteration in GCNs constitute a driving force to adaptation of cellular metabolism from one steady state to another, frequently in response to environmental changes [60,61]. These events resemble the network rewiring of health-disease transition [62]. The GCNs for the Caco-2/EH41 group displayed an anomalous degree distribution regime after one hour of cell–bacteria interaction, the persistence of this condition along the second hour, and the establishment of a new gene hierarchy thereafter. The new established network topology represents an adaptive cell response to the pathogen, not a return to the “normal” state. Conversely, the networks for Caco-2/Ec472 group showed a slow and progressive tendency to an anomalous degree distribution regime, with no topology restoration until the end of the exposure interval here studied. Because these networks do not present large maximum degrees, tests such as Kolmogorov–Smirnov [15] cannot be directly applied to ascertain their scale-free nature. However, it is clear that the initial stages of the studied GCNs are more compatible to the scale-free behavior than in the case of the anomalous distributions found for the networks at later intervals. These results revealed that EH41 and Ec472/01 strains induce very distinctive enterocyte responses, reflected as pronounced differences in network topology.

Enrichment analysis based on KEGG pathways for the DE gene set revealed that Caco-2/EH41 has many genes involved in the inflammatory response, where NF-Kappa B, TNF, and RIG-I-like pathways are significantly represented (Figure 3). Other DE genes were involved in different biological processes, such as cytoskeleton organization, phagocytosis, apoptosis, and adaptive immunity. This scenario was already observed in our previous study where the gene–gene network of Caco-2 cells exposed to EH41 presented hub genes were involved in the activation of inflammatory response and neutrophil recruitment via TNF signaling pathway [7]. Moreover, SEM images showed that changes of epithelial cell morphology are clearly more severe after cell exposure to EH41, including brush border and microvilli destruction (Figure 8). These results showed, for the first time, how rapid are the Caco-2 cells’ genomic and phenotypic alterations induced by EH41 compared with induced by Ec472/01.

Therefore, we investigated how these pathways and phenotypes are induced in Caco-2 cells along three hours of interaction with STEC. In GCNs, the transcriptional modules encompass genes presenting high co-variation expression across the samples and their expression profile pattern are distinct from those of the other modules [17]. Hence, transcriptional modules correspond to a group of genes functionally linked, which play specific biological functions and/or belong to a common molecular pathway [17,63,64,65].

Here, we used the WGCNA approach for associating transcriptional modules with three sequential time-intervals juxtaposed windows (JW1, JW2, and JW3). The enrichment analysis of modules that are highly correlated with each window revealed the molecular mechanism dynamics of Caco-2 differential response to the two STEC strains. EH41 induces a rapid inflammatory and apoptotic response in Caco-2 just after the first two hours of enterocyte–bacteria interaction and, in the third hour, just a few hubs were still involved in the cell response to the bacteria (Figure 6c). Conversely, Ec472/01 elicits a Caco-2 response where the GCN’s high-hierarchy genes (hubs) are mostly involved in cytoskeleton organization along the first two hours of response, and just a few hubs appear involved in inflammatory and adaptive immune responses. Additionally, along the third hour of bacteria–enterocyte interaction, many hubs in the Caco-2/Ec472 network were predominantly associated to innate immune response modulation. Here, it is worth mentioning previous work providing evidence that acute inflammation plays a role in the development of HUS [66,67]. Patients with HUS have a rise in C-reactive protein, neutrophilia, and an increase in circulating proinflammatory cytokines, indicating that the impact of hemorrhagic colitis may be important for the subsequent development of severe complications, such as HUS and encephalopathy [66,67].

Finally, SEM phenotypic analysis of Caco-2 cells along enterocyte–bacteria interactions showed more intense microvilli destruction in cells exposed to EH41, when compared to cells exposed to Ec472/01. Both bacterial strains induced in Caco-2 cells showed the expression variation of many genes encoding for cytoskeleton structural molecules or involved in cytoskeletal organization (Figure 3). However, in the Caco-2/Ec472 experimental group, more genes and hubs were involved in cytoskeletal organization compared with the Caco-2/EH41 group. This result points to our finding that EH41 cause intense microvilli destruction and, consequently, indicates that such processes contribute to bacterial colonization, since the microvilli establish an electrostatic barrier to microbial adhesion [68].

## 5. Conclusions

We employed dynamic gene co-expression network (GCN) analysis in order to investigate the differential response of Caco-2 cells to two STEC strains, one patient-isolated and associated with HUS (EH41) and the other (Ec472/01) isolated from cattle feces in Brazil. The integration of GCN and phenotypic data allowed us to conclude that EH41, comparative to Ec472/01, induces greater and earlier global gene expression alterations in Caco-2 cells, which is related to excessive inflammatory and apoptotic responses. These responses are associated with the pronounced morphological alterations observed by SEM in Caco-2/EH41. Altogether, these results show that EH41 expresses virulence genes, inducing a distinctive host cell response and is likely associated with more severe pathogenicity. In order to better understand the mechanisms involved in STEC pathogenicity and discover markers for potentially highly pathogenic STEC strains, further studies on this subject should focus on: (i) the STEC disease-associated module genes and molecular pathways involved in the induction of immune responses leading to severe conditions, such as HUS: (ii) the influence of environmental factors on STEC virulence, including the passage through bovine and human digestive tracts.

## Figures and Tables

**Table 1:** Hubs in Caco-2/EH41 network.

Gene	Module	Gene Ontology	Functional Description
*MEG3*	Blue	Long non-coding RNA	apoptosis
*ROPN1L*		epithelial cilium movement	epithelial cilium movement
*CSH1*		JAK-STAT signaling pathway; PI3K-Akt signaling pathway	inflammatory response
*ADRA1A*		positive regulation of ERK1 and ERK2 cascade	inflammatory response
*TBKBP1*		RIG-I-like receptor signaling pathway	inflammatory response
*NEGR1*		protein binding	protein binding
*P2RX2*		positive regulation of calcium-mediated signaling	signaling
*FLJ40434*		ND	unknown
*LOC157740*		ND	unknown
*BBC3*	Turquoise	Hippo signaling pathway	apoptosis
*UFC1*		response to endoplasmic reticulum stress	apoptosis
*YY1AP1*		regulation of cell cycle	cell cycle
*DOK1*		Ras protein signal transduction	immune response
*IRF1*		positive regulation of interferon-beta production	inflammatory response
*TICAM1*		positive regulation of I-kappaB kinase/NF-kappaB signaling; Toll-like receptor signaling pathway	inflammatory response
*BCL3*		I-kappaB kinase/NF-kappaB signaling; TNF signaling pathway	inflammatory response
*TNFRSF1A*		positive regulation of I-kappaB kinase/NF-kappaB signaling; TNF signaling pathway	inflammatory response
*BIRC3*		positive regulation of I-kappaB kinase/NF-kappaB signaling; TNF signaling pathway	inflammatory response
*MAFF*		transcription from RNA polymerase II promoter	transcription
*RG9MTD1*		positive regulation of mitochondrial translation	transcription
*NUP62*	Brown	negative regulation of apoptotic process and programmed cell death	anti-apoptosis
*PARL*		negative regulation of intrinsic apoptotic signaling pathway	anti-apoptosis
*NASP*		DNA replication-dependent nucleosome assembly	cell division
*HIST1H1C*		chromatin DNA binding	cell division
*C1orf96*		regulation of mitotic spindle assembly	cell division
*HIST1H2BH*		chromatin organization	chromatin organization
*SAFB*		chromatin organization	chromatin organization
*MARCKS*		actin filament bundle assembly	cytoskeleton
*HIST1H2BJ*		LPS binding	immune response
*SLC25A22*		L-glutamate transmembrane transport; mitochondrial transport	metabolic process
*HIST1H3H*		chromatin organization	transcription
*HIST1H4K*		telomere organization	transcription
*HIST2H3A*		chromatin organization	transcription
*NUP153*		protein sumoylation	transcription
*DNMT1*		transcription	transcription
*UBE2Q1*		protein ubiquitination	ubiquitination

ND, not determined.

**Table 2:** Hubs in Caco-2/Ec472 network.

Gene	Module	Gene Ontology	Functional Description
*CENPN*	Yellow	mitotic cell cycle	cell cycle
*CHAF1A*		DNA repair	cell cycle
*ZWINT*		RHO GTPase Effectors	cell division
*CDCA5*		mitotic cell cycle	cell division
*RAD51*		DNA repair	cell division
*WDR62*		RNA splicing	cell division
*CCNA2*		Ras protein signal transduction	cytoskeleton
*EMP2*		actin filament organization; cell adhesion	cytoskeleton
*PODXL*		regulation of microvillus assembly	cytoskeleton
*KCNN4*		positive regulation of T cell receptor signaling pathway	immune response
*E2F2*		transcription factor	transcription
*UBE2T*		DNA repair; protein ubiquitination	ubiquitination
*PRIMA1*	Black	cell junction	cell adhesion
*ITGA9*		cell adhesion; PI3K-Akt signaling pathway	cell adhesion; immune response
*KRT9*		structural constituent of cytoskeleton	cytoskeleton
*RAC2*		actin cytoskeleton organization; Chemokine signaling pathway	cytoskeleton; inflammatory response
*KRTAP10-10*		keratin filament	keratinization
*C8orf73*		alias *MROH6*; lipid transport	lipid transport
*AQP5*		microvillus; Aquaporins	molecular transport
*ANKRD13B*		endosome	protein transport
*HAX1*	Turquoise	interleukin-1 binding; regulation of apoptotic process	anti-apoptosis
*SSNA1*		G2/M transition of mitotic cell cycle	cell cycle
*UPF1*		DNA repair; cellular response to LPS/interleukin-1	immune response
*TRAFD1*		negative regulation of innate immune response	immune response
*DOK4*		protein binding; negative regulator of T cells	immune response
*ACOT8*		acyl-CoA hydrolase activity	metabolic process
*STX5*		SNARE binding; vesicle-mediated transport	protein transport
*COPZ1*		vesicle-mediated transport	protein transport
*WBP1*		WW domain binding	protein binding
*NOTCH2NL*		Notch signaling pathway	signaling
*TMEM116*		transmembrane protein	unknown

## Supplementary Materials

The following are available online at https://www.mdpi.com/2076-2607/7/7/195/s1, Figure S1: Analysis of network topology for Caco-2/EH41 and Caco-2/Ec471 groups. A and c—graphs of scale-free fit index (y-axis) as a function of the soft-thresholding power (x-axis). B and d— graphs of the mean value of the node degree (y-axis) as a function of the soft-thresholding power (x-axis); Figure S2: Hub categorization. Scatterplots between network connectivity (x-axis) and intramodular connectivity (y-axis) of the genes present in blue, turquoise, and black modules of the Caco-2/EH41 group (column a), and the genes present in yellow, black, and turquoise modules of the Caco-2/Ec472 group (column b). The dot colors correspond to modules and the star dots identify selected hubs; Figure S3: qPCR technical validation of DNA microarray gene expression data for Caco-2/EH41 group; Figure S4: qPCR technical validation of DNA microarray gene expression data for Caco-2/Ec472 group; Table S1: Primers for qPCR technical gene expression microarray data validation; Table S2: KEGG enrichment analysis for the DE genes (Venn diagram) in the groups Caco-2/EH41 and Caco-2/Ec472; Table S3: KEGG enrichment analysis for the nine selected modules of Caco-2/EH41 network; Table S4: Hubs in Caco-2/EH41 network and their gene expression profiles across time interval juxtaposed windows; Table S5: KEGG enrichment analysis for the nine selected modules of Caco-2/Ec472 network; Table S6: Hubs in Caco-2/Ec472 network and their gene expression profiles across time interval juxtaposed windows.
